# A Node Detection Method Based on Johnson–Cook and Thin-Film IMD Characteristic Model Armor Damage Detection Repair and Subsequent Optimization

**DOI:** 10.3390/polym14214540

**Published:** 2022-10-26

**Authors:** Hanjui Chang, Guangyi Zhang, Yue Sun, Shuzhou Lu

**Affiliations:** 1 Department of Mechanical Engineering, College of Engineering, Shantou University, Shantou 515063, China; 2 Intelligent Manufacturing Key Laboratory of Ministry of Education, Shantou University, Shantou 515063, China

**Keywords:** armor node, detection of battle damage, detection accurate repairs, position prediction

## Abstract

In this paper, a node detection method is proposed for the detection of battle damage to armor. This experiment uses the special nature of the film to virtualize the surface of the armor IMD film coverage. The die index is a large area and is easy to damage, but with the use of a unique IMD film stamping die, the possibility of damage decreases, which provides a damage prediction function for the armor. In addition, for the damaged armor, the same method can be used to detect because the damaged part more easily causes the surface film to rupture after being impacted, so it is possible to optimize the design of the armor and the molding through the die index. The die index can also detect the degree of damage to the damaged part of the damaged armor. Therefore, the IMD die index is introduced to quantify the data, and the degree of damage is judged by the IMD die index. The novelty of this work is that each node can efficiently detect the vulnerable damage position of the armor using the die index and then pass through the COMSOL. The Johnson–Cook stress model simulates the battle loss, obtains the stress deformation that occurs after the battle loss, and verifies the experiment by comparing the results obtained. Finally, the repair method is used to repair all the predicted battle damage parts based on additive manufacturing to ensure that they can be used again after repair.

## 1. Introduction

Polymer materials are the most attractive materials for armor ballistics currently being developed. Polymer is a new raw material used in the production of composite bulletproof covers and armor, such as shrapnel and bulletproof helmets, armor, explosion-proof devices, and inserts in bulletproof vests, generally using heterogeneous material structures. Polymer materials can be composed of metals and polymers, and new materials with higher impact strength and better energy absorption can be obtained by combining different materials with the desired properties. These properties are greatly compromised if the composite exhibits defects in interlayer separation, bubbles, or delamination, which are usually caused by in-mold impacts during the molding of the composite model. This paper presents an IMD thin-film model of a composite material that introduces defects and performs a series of mold flow software simulations to locate the composite. The location of the model is prone to defects, so the prediction of overlay damage can become quite simple, which is the overall efficiency improvement brought about by this method, and the repair process is planned from the recovery of the damage. The filling of armor defects in 3D-printed materials is carried out in the pad, and the effect of reinforcing the strength of the overall composite armor is achieved by repairing part of the pad. For example, the cover is added to the armor panel by laminating as well as vacuum bags to provide proper compaction pressure. Secondary repair and tertiary repair require more material repairs, which are still under study. They combine the tensile properties, thickness, and bending properties of the diaphragm and the elongation properties of the circuit. When setting its parameters, pay attention to the impacts of the shape of the diaphragm, the corner radius of the convex mold or the convex die, the edge pressing force, the gap in the concave and convex die, etc. on the tensile depth. In order to avoid the deformation of the product, when designing the film, its wall thickness must be consistent. When solving the heat retention temperature caused by the plastic diaphragm, the temperature difference in the heat transfer can be calculated after cooling analysis, and the mold temperature on the die side can be compensated to minimize its deformation.

In previous studies, the traditional bullet detection method is that armor or body armor is divided into multiple areas. If necessary, you can obtain higher accuracy by increasing the number of partitions and reducing their coverage. Several areas are covered by an irregularly arranged cable, and any penetrating bullet cuts a part of the cable. One end of the cable is grounded, and the other end is connected to the input of the microcontroller. The input of the microcontroller is dedicated to distinguishing between multiple regions and creating an interruption when the cable is cut by a bullet, and when the cable is cut, a particular input will receive a different voltage and current signal.

In the armor system, the fiber diameter, material strength, and bulk density of the composite polymer material are the key parameters in this study and in previous research, and the optimization of these parameters will have important impacts on the defect indicators of the finished product after the subsequent armor molding. What has been overlooked in previous studies is that injection-molding defects generally exist between various products, and if the products come from different suppliers, the vulnerable parts of the armor obtained will be different, such as in this typical instance: There are 150 separate layers in a ballistic armor system, and each injection-molded part interface between each layer affects the ballistic performance of the system in some way, including friction, sliding and sandwich surfaces, air gaps of approximately 0.05 mm–0.1 mm that may form within and between injection-molded components and carriers, and interconnection spaces between soft armor components. The geometric coordination between stacked shields has an impact on traditional armor prediction.

**Figure 1:**
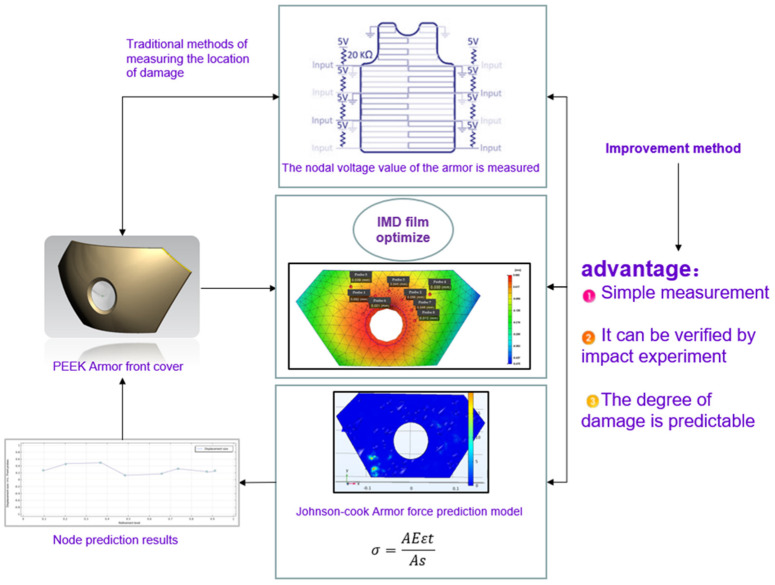
Thin film IMD characteristic model test steps and advantages.

Generally, on the surface of the damaged armor, it is necessary to carry out the corresponding detection operation at the damaged interface in time, obtain the corresponding voltage, and then conduct a certain comparative analysis to obtain the detection results. This approach still produces a certain prediction bias and does not necessarily reflect the flaws of the armor very intuitively. The novelty of this paper can be summarized in Figure 1.

## 2. Highlight

- Each die node can efficiently detect the location of the damage to the armor through node detection, so that subsequent accurate repairs can be carried out, and the number of areas and nodes can be changed as needed for more accurate position prediction.
- Based on the die index node analysis of IMD films, we use this technique in armor damage prediction because the die defects in the film are very similar to the surface damage defects in the armor and can be analyzed as the same defect factor.
- Factor analysis and the improvement of part defects, IMD analysis results for armor parts before and after optimization, mold indices before and after IMD mesh analysis, and film parameter optimization are used to optimize armor damage defects.
- Finally, battle loss simulation is carried out with the Johnson–Cook stress model of Comsol, the stress deformation after the battle loss is obtained, and the experiment is verified by comparing the results.

## 3. Literature Reviews

In the overall system of armor or body armor, due to differences in materials and molding methods, previous studies have basically regarded the fiber diameter, the strength of metal surface stiffness, the density of woven fabric, and the density of glass fiber as very important. The conditions for the key research parameters, including monitoring and adjusting these parameters, will form the basis of the quality control system as well as the regulatory system in the study, for example in the highly compressed ultra-high-molecular-weight polyethylene and variants of this material, before the interface of the multilayer backing layer and its adjacent layer.

In 2008, Chen Shizhong et al. [1] separated PC film surfaces from the grid, and the results showed that the mold temperature had the most significant effect on the tensile ratio and the rate of change in film thickness. Size and thickness changes are not sensitive to film thickness, and multistep thermoforming has a certain effect on reducing the warpage and folding of composite films. In 2008, C.O. Phillips [2] studied the material properties of composite films for in-mold decoration (IMD) made from a mixture of PBT and polyethylene terephthalate during the injection-molding process. The results show that greater molecular flexibility reduces the transition temperature of the amorphous glass phase. In 2009, Gugyong Kim et al. [3] proposed a method for determining Gsell parameters using a single-axis tensile test at constant crosshead velocity to predict IMD film thickness distribution and printed pattern variations. The results show that when using a multicavity mold system, the groove arrangement can be controlled to obtain a better uniform distribution of IMD film quality and pattern. In 2010, Shia-Chung Chen et al. [4] studied IMD film molding in the shape of a mold cavity and found that heat transfer on the cavity side was hindered during IMD molding. Studies have shown that under the conditions of injection molding, coolant temperature, melt temperature, IMD film material and film thickness, and other injection-molding parameters, when the thermal conductivity of polycarbonate film increases, the initial cavity temperature may affect the melt–film interface temperature when the melt is in contact with the film, and this temperature will change with the film cavity. In 2010, Shuai Zheng et al. [5] discussed the processing of the overall armor plate of fiber-layered polymer materials, the evaluation of ballistic performance, the demonstration of repair strategies, and the update of ballistic performance after repair. The results show that the impact of reimplanted layering on the trajectory performance during repair is small, which proves the robustness of the armor design and subsequent repair. Compared with baseline repair, the ballistic performance of the panel repair of the replacement tile can be guaranteed.

In 2011, A. Martinez et al. [6] studied the characteristics of different textiles used in the pressure drop process that affect the padded part of the material. The results show that the pressure drop of plastic melting and gluing flowing through the textile when using the spiral mold is 15% higher than that obtained during conventional injection molding, and the specific reason is that the polymer material must compress the textile foam in addition to the compression caused inside the mold. In 2020, S. Mahdi et al. [7] investigated a new type of composite structural armor that provides ballistic protection and sufficient strength at minimum weight. The results show that the room-temperature curing armor repair system only provides limited ability to bond to different composite layers; thus, low-temperature curing conditions provide enough interface strength for the armor repair system to allow armor repair to restore the original structural performance.

In 2018, Mulat Alubel Abtew et al. [8] studied the effects of different stitching parameters on the formability of multilayer composite armor, and the quilting of stitched diamond-shaped materials showed very few tensile values at both latitude and longitude. In the experiment, the material deformation recovery rate of unstitched and diamond-quilted prefabricated parts was the highest and the lowest, respectively. The results show that with the reduction of the sewn gap and suture length of the composite material, the deformation recovery rate of each layer of armor becomes very low. In 2020, Mawkhlieng et al. [9] studied the effects of material and structural parameters for the use of new fibers in soft armor. This included the implementation of 2D, 3D, 3-axis, and knitted fabrics to enhance structural integrity. For impact, the results show that the curl-free UD fabric structure dissipates stress waves faster and more widely. In 2020, Ian G. Crouch [10] studied the parametric relationships between fiber diameter, fabric density, and ceramic density in armored systems, and by monitoring the relevant quality control procedures composed of these parameters and the interfacial properties between adjacent layers of multilayer backing panels, the results showed that the size of the sandwich gap between fiber substrates and the geometric fit accuracy between hard armor plates in stacked-armor systems are particularly important for the quality molding process.

In 2021, Muhammed Kamrul Islam et al. [11] reviewed various forms of structural design strategies by imitating natural strategies, such as layered structures, overlapping structures, and double-layer structures. The research work outlines the biocontainment mechanism and technology design bionic structure, and the results will help subsequent researchers to build new material armor and structures. In 2020, Sangeeta Khare et al. [12] used the Johnson–Cook model to make predictions about the flow of material from the armor plates. The damage parameters were determined in conjunction with the simulation. The authors simulated different notched specimens between certain ranges of radii under tensile loads. The results show that the load–strain curve is well matched with the simulation experimental data. In 2021, Xiaotian Li and Murilo Augusto [13] proposed a stress analysis model for estimating the number of bending cycles required to induce lateral buckling under wet epic conditions. The calculations show a relatively good correlation between buckling and the number of bending cycles. Based on this analysis model, the effects of the axial compression performance, bending curvature, and coefficient of friction on the number of bending cycles and the stress state at the beginning of buckling are discussed. In 2021, Aayush Bhat et al. [14] researched the material selection and analysis of armored systems and discovered important material properties suitable for ballistic applications. Multilayer fiber armor improves the overall safety of the armor by ensuring that the impact absorbs a large amount of kinetic energy, and the results show that the use of natural glass fibers in polymer materials can provide better ballistic performance. In 2022, Han-Jui Chang et al. [15] proposed optimizing the quality of drone shell parts through multiobjective optimization. According to the comparative analysis of experimental verification and simulation data, it is concluded that injection-molding time and pressure time are the most strongly correlated with mold indicators. Therefore, by improving the injection time and pressure, and using multiobjective optimization to obtain the optimal combination of injection parameters to improve the IME film quality of the die process, the molding quality of the core chip area of the UAV is improved.

In 2022, Han-Jui Chang et al. [16] used a multiobjective optimization method to optimize automotive pedal injection-molding defects. The optimized injection-molding parameters are cycle time and warpage, and the Pareto boundary of multiobjective optimization results is used to identify the nonlinear relationship between cycle time and warpage to find the Pareto optimal solution of warpage values under different cooling times. Finally, experimental images of multiobjective optimization results in line with actual production requirements are provided under four injection-molding influence parameters. In 2020, Sang YoonLee et al. [17] studied the highly ductile conductive ink of the three-dimensional in-mold electronic technology IME, and the results achieved the injection and installation of electronic circuits and optical devices; after forming a tensile conductive ink, it could precisely display printed circuit patterns and optimize the injection-molding process. After high temperature and high pressure, the ink filler is tightly filled, resulting in a significant improvement in the conductivity of the ink. In 2020, Yao Gong et al. [18] studied how the resistance changes with the deformation of the printed circuit during the IME thermoforming process. The deformation defects of the printed circuit are compared with the numerical results of the simulation, and then, according to the established numerical simulation results and regression model, the resistance distribution and the amount of resistance of the printed circuit after the thermoforming process can be accurately predicted. In 2020, WeiGuo et al. [19] combined microporous injection molding with in-mold molding, and the simulation found that by changing the heat transfer ratio in the MIM mold, it was possible to simulate foam parts with better appearance. By comparing the simulation results with the experimental results, it was found that the established response model could accurately predict the temperature field of the IMD molded parts.

Therefore, the results show the effect of the film on the crystallization phenomenon of IMD-molded parts. In 2020, Wu Chengxuan and others [20] designed a series of new film molding and injection-molding experimental methods that break with the traditional one. Some of these thermoforming and molding process quality-influencing factors are not considered for experiments; only mold temperature, melt temperature, injection speed, and packing pressure are used as the injection parameters for research. Some of the injection-molding process parameters were further optimized using the Taguchi experiment, and the factor that contributed the most to the results was the packing pressure. Therefore, the conductive wire in the finished IMD mold is locally hot pressed, and the finished product resistance measured after each process is almost the same, meeting experimental requirements. In 2021, Liu Xiangyang et al. [21] proposed a new method of IMD ink treatment based on broadband ultraviolet light, which can form traces with excellent electrical properties compared with traditional hot sintering, while the production time is longer than the traditional method. Ink UV treatment provides a new platform for the rapid production of conductive silver from silver oxalate molecules on substrates and the development of 3D interfaces that break with traditional thermoforming. In 2021, Mehdi Moayyedian et al. [22] used a combination of neural networks and Taguchi experiments to solve a set of solutions for the optimal parameters of injection molding. The weight of each defect factor of the thin-walled part was calculated by layering analysis to simulate the molding process of the polypropylene injection-molded part, and the proposed optimum was verified experimentally. The combination of injection-molding parameters meets the actual requirements of the part. The result is that filling time has the most significant impact on finished product defects and molding quality. In addition, the error margin of the uncontrollable injection-molding parameters during the injection-molding process is 1.5%. In 2021, Han-Jui Chang et al. [23] proposed a screw-life prediction model of hybrid composite screw process parameter method. In order to meet the requirements of efficient production, a combination of an automated virtual metering system and a recognizable performance evaluation program is proposed. The method predicts the injection of screws by extracting a combination of cutting conditions and related process parameters from available sensor data, taking sampling inspection methods with measurement delays from real-time and online routine inspection procedures.

The above study analyzes hybrid panels composed of various polymer materials to provide better composite ballistic strength located on the impact surface. Although the new materials can well replace the ballistic impact performance of traditional hardware armor, the cost and moldability of composite materials still need to be studied in depth. Lightweight metals provide a low-cost, high-return solution to the problem of the basic production of armor. For example, aluminum gold provides a suitable substitute for the manufacture of low-cost armor at a lower price that has good material mechanical strength and impact resistance. Compared with other monolithic materials such as steel, aluminum alloys have been shown to have greater material energy absorption and material permeation resistance, undergoing quenching. The high toughness and integrity after the molding stage and tempering stage make the aluminum alloy a good material for traditional body protection. On the other hand, the hardness of steel is also a determinant of ballistic performance, and it can also be used for protective bodies due to the penetration resistance and light weight of steel armor material. Magnesium alloy has high yield strength and ductility; although the hardness is weaker than that of aluminum alloy, the density of magnesium is lower, so the traditional previous research has also studied the use of magnesium as a lightweight alternative to aluminum. At the same time, magnesium alloy materials also have high specific damping, becoming one of the powerful materials for energy absorption and shock absorption. However, the alloy material is still generally very heavy and not suitable for long-term combat wear; the impact resistance of the existing new glass-fiber armor can be attributed to the high compressive strength and fracture toughness between the glass fibers, so in order to fuse the above alloys’ performance, this article refers to the use of an armor made of tough materials such as composite glass-fiber PEEK laminates to prevent bullet fragments passing through the armor. In particular, glass-fiber materials are used as impact surface materials. If the thickness of the impact surface of the glass-fiber material is reduced to some extent, the thickness of the backing surface needs to be subsequently increased to maintain the penetration resistance of the material. In addition, the Johnson–Cook flow stress and Johnson–Cook failure models predicted the material flow behavior of the armor plate and proved to be effective methods. This method, combined with simulation, can determine the damage parameters of the model.

## 4. Methodology

The advanced PEEK glass-fiber material used in this experimental armor is a very promising material, combining the advantages of high strength and ductility as well as excellent impact resistance and good formability. PEEK materials may experience various changes in strain rates and pressure temperatures when subjected to a wide variety of dynamic load conditions, such as ballistic shocks.

In order to predict the behavior of PEEK materials under these different factors, it is first necessary to study their flow stress and failure initiation behavior experimentally and incorporate them into the law of failure. Modeling and predicting the flow behavior of materials using Johnson–Cook flow stress failure models has proven to be an effective method. This method can be combined with injection-molding simulation to determine the damage parameters of the model. Therefore, the JC model is one of the widely used models for predicting the dynamic behavior of glass-fiber materials. Johnson–Cook’s flow stress model, however, depends on different states of strain, the magnitude of the strain rate, and changes in temperature. Usually in large strains, the material fails due to damage to the ductile material due to the void in the material layer: the final void merger leads to the failure of the material. Failure-induced structural changes are predicted by a separate failure model that associates this strain with related parameters such as triaxiality in the material.

Since the JC failure model is exponentially dependent on the triaxiality of the force, it is usually necessary to study it by performing tensile tests on round rod specimens of PEEK materials with different notch radii. In general, the overall distribution of strain states is almost uniform in the neck, while the center of a few notches is triaxial and always occurs at the notch. Predecessors provided the triaxial equation for the center of these specimens, where R is the radius of the curvature of the neck and the radius of the neck segment is the radius of the neck. The initial stress triality of the specimen is 1/3, and the notches of different radii have different triaxiality. Thus, the equation predicts the higher stress triaxiality of the generally lower radius notch. The equivalent plastic strain at the fracture of PEEK materials is given as

(1) $$\varepsilon_{f}=2In\left(\frac{d_{0}}{d_{f}}\right)$$

where $d_{0}$ and *d_f_* are the initial diameter of the part and the diameter of the fractured surface, respectively, but the triaxial effect formula of the model is limited to round products and cannot predict the triaxiality of flat products, such as hot-rolled sheets and the front cover of the armor. This sheet is widely used in the automotive industry, aerospace engineering, and defense applications.

(2) $$\eta =\frac{1}{\sqrt{3}}\left[1+2\ln\left(1+\frac{t}{4R}\right)\right]$$

Since few studies have used planar specimens to study the triaxial effect, this paper uses the triaxiality of the center of the gap area of the plane–strain specimen proposed in the latest previous research, where R is the notch radius and t is the thickness of the specimen at the notch area The triaxiality of the flat interview sample of the gap should be ≥1/√3. The length f of the neck segment of the flat specimen in a section of flat was given as

(3) $$\varepsilon_{f}=\frac{2}{\sqrt{3}}In\left(\frac{t_{0}}{t_{f}}\right)$$

where $t_{0}$ and *t_f_* are the initial material thickness and material fracture thickness at the groove on the armor damage surface, respectively. From the experience of previous generations, it is known that it is very difficult to measure the thickness *t_f_* of the gap at the site of injury failure and that calibrating the JC model may lead to erroneous injury site predictions. Therefore, the more advanced method is to determine progressive strain R in the fractured area of armor damage with the area reduction method.

The Johnson–Cook flow stress failure model is used to predict the material behavior of glass fibers. The glass-fiber PEEK material used in this study is a 3 mm thick armor front cover plate that makes a flat stretch according to the standard geometry. The armor experiment subject, which then tests the subsequent engineering stress (*σ_e_*) and engineering strain (*ε_e_*) on the subsize of the geometry of the armor part. It can be obtained by measuring steady-state loads and steady-state displacement responses by extensometers:

(4) $$\sigma_{e}=\frac{F}{A_{0}}$$

(5) $$\varepsilon_{e}=\frac{\Delta L}{L_{0}}$$

(6) $$\sigma =\frac{AE\varepsilon t}{As}$$

*F* is the force, *A*_0_ is the area of the cross-section of the specimen, and Δ*L* and *L*_0_ are the changes in the specimen length and the original gauge length, respectively. The true stress or true strain are calculated as follows:

(7) $$\sigma_{t}=\sigma_{e}\left(1+\varepsilon_{e}\right)$$

(8) $$\varepsilon =\frac{2C0}{ls}\varepsilon_{r}dt$$

(9) $$\varepsilon_{t}=In\left(1+\varepsilon_{e}\right)$$

*A* and *E* are, respectively, the cross-sectional area size and elastic modulus values of PEEK glass-fiber material armor. *C*0 is the speed of sound wave propagation in the strip-shaped material, and then As and ls are the cross-sectional area and length of the armor model, respectively. Equations (8) and (9) give engineering stress and strain values for a range of stress conditions that will be converted into the true stress and true strain of the armor under real conditions following the JC experimental empirical numerical method. Equations (6) and (7) can be compared optically by comparing sequenced images during specimen loading, providing precise displacement and strain fields on the damaged surface of the armor. Only one sensor is required for in-plane measurements, but two sensors are required for out-of-plane displacement. The equation also takes into account the relative displacement outside the plane of the failure region of the armor stress calculation, so that the strain of the central area of the gap can be provided.

Therefore, when designing the armor structure before the experiment, the material behavior data of the glass-fiber material under deformation and high strain during the loading process should be obtained. In this experiment, the combined simulation of the material flow behavior of glass fiber based on the Johnson–Cook failure model is used to model and predict. The material parameters of PEEK flow stress are the PVT plots of the temperature and material determined by tests performed in a certain strain rate range. Therefore, the damage parameters of the material failure model are proposed to be determined by combining the calculation parameters of the model specimen with the injection-mold flow simulation. Using these material parameters, the degrees of depression under steady-state loads and dynamic impact are, respectively, 0.05 mm and 0.1 damage armor parts with different notches of three levels were rated by nodal damage tests.

## 5. Case Study

The purpose of this experiment is to predict the locations of defects in armor, but traditional methods are generally achieved by experimentally verifying that the voltage system is working properly according to the required specifications. Once the wires are cut by a bullet, the controller creates a voltage change indicating that the vest has been hit. After reading the degree of voltage change indicated by the voltmeter, and by sensing that the node voltage is lower than the normal range—proving that there is damage in the area—the degree of damage needs to be further ascertained. This method faces the problem of complex steps, long time consumption, and excessive cost, and it cannot achieve molding defect prevention in the armor. By analyzing the die index of the node position, the two main problems faced by real-time defect prediction and the prevention and reinforcement of the defective parts of the molding can be solved, and efficient simulation can be realized, eliminating many tedious experimental steps in practice.

Polymer-composite glass-fiber materials in the study of armor material selection play an important role in absorbing energy because of their high toughness and light weight. An important feature of this advanced composite is that the flow volume is temperature-dependent, so the desired performance can be obtained by adjusting the temperature, which may affect the ballistic properties of the material. The carrying capacity of composite glass-fiber materials depends on the type of fiber and, more importantly, the orientation of the fiber layer. When the load acts in the direction of fiber laying, the impact strength of the fiber is often the strongest. The impact results in the generation of longitudinal and shear waves in the fibrous layer, which helps to detect areas of failure. In addition to energy absorption through failures such as fiber shear blockage and fiber delamination, the partial cracking of the matrix is also one of the ways of energy absorption. Low-speed and high-speed impacts produce different forms of damage to the laminate of glass-fiber materials. In low-speed impact, the armor structure support conditions play a major role, and the support conditions have less impact on high-speed impact than the materials. Therefore, it is only necessary to focus on the selection of materials, explore the strength properties of materials with different fiber densities, and determine the specific data of the materials used as PEEK material PVT diagram describes.

**Figure 2:**
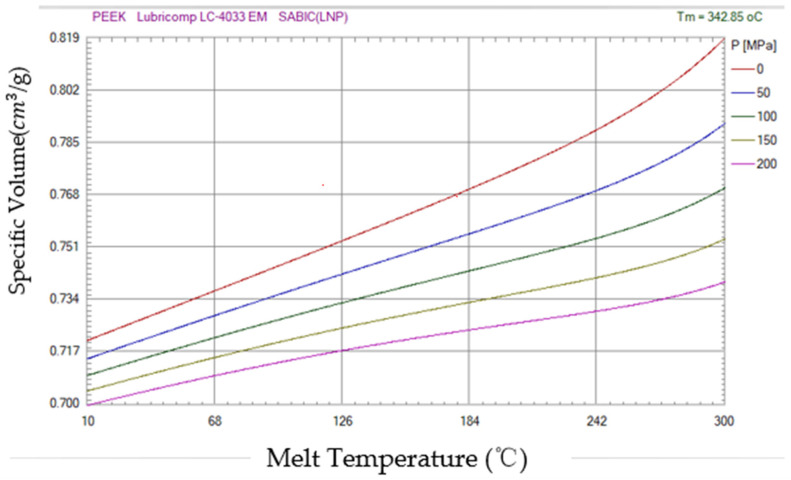
PVT diagram of PEEK material.

In terms of materials, the PEEK plastic film used in this experiment has the characteristics of good high-temperature dimensional stability, high transparency, high temperature resistance and friction resistance, and small shrinkage. According to the three-parameter solid model of the composite material in the previous research, the creep characteristics and stress relaxation of the PEEK polymer can be reflected, and the viscoelastic plastic model of the PEEK diaphragm is theoretically constructed. Figure 2 presents a PVT diagram of PEEK material for characterizing the elastic plastic portion of the PEEK diaphragm during deformation. The thickness of the material used is between 1.59 mm and 5.04 mm.

**Figure 3:**
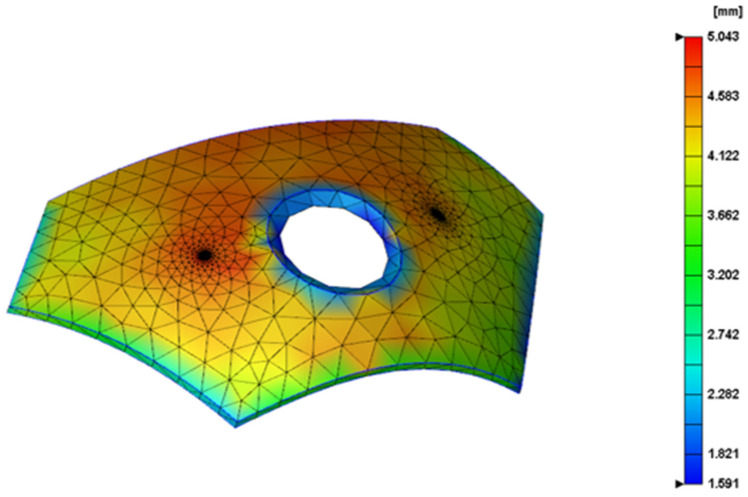
Peek material armor thickness distribution map.

In the research on the molding quality of PEEK armor and film, the molding of plastic films is first involved in the process of the in-mold IMD of the surface of the injection-molded products. The PEEK thin film in the experiment is first placed in the mold cavity before molding, and then the polymer material, which is also PEEK, is melted into the mold cavity and bonded to the film. After the molded film leaves, the carrier film can be permanently adhered to the surface of the plastic product. When the melt cures, the mold can achieve a better decorative effect after rejecting the product, and the carrier will automatically peel off while preparing for the next production cycle. In the mold design, the film and the injection mold cavity should cooperate with each other: the size of the two should have high processing accuracy to match. According to the extension ability of the film itself, pay attention to keeping the film out of the turning position of the sharp the location. The PEEK material armor thickness distribution map is shown in Figure 3.

**Figure 4:**
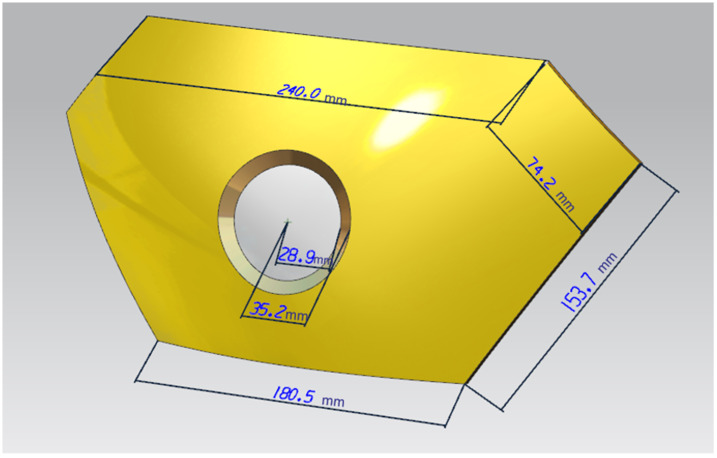
Exterior dimensions of the armor.

First, the damaged armor is 3D scanned to obtained the 3D parts we need, as shown in Figure 4, and then, according to this experimental method, the detection node is set up, and the damage site is identified by the current and resistance between the nodes; the armor will be divided into opposing nodes. The more complex approach of the traditional method is that each area is covered by a wire, so any penetrating bullet will cut the wire. The wires are connected to the node end via a pull-up resistor. The inputs of the control node will distinguish between regions and receive the set voltage when the wire is cut, and fluctuation in the resistance occurs.

This experiment will use the special properties of the film to cover the surface of the armor with a virtual IMD film. Using IMD, the unique nature of the film die makes it easy to crack and puncture the damage area. The area with a large die index is easily damaged, and the possibility of damage is improved, which provides a damage prediction function for the armor. For the damaged armor, this method can also be used for detection because the damaged part is more likely to cause the surface film to rupture after the impact; therefore, both the armor design and molding optimization can be carried out through the die index. The damage to the armor can be detected.

Before repair, the damage status of the armor is assessed: Damage depth less than 0.1 mm is classified as level 3 damage, damage between greater than 0.1 mm and the shooting through wire is level 2 damage, and the direct injection through wire is level 1 damage. Judging and evaluating based on the current and resistance values obtained by the detection, the first two degrees of damage can be repaired, and the last degree reaches the scrap standard.

**Figure 5:**
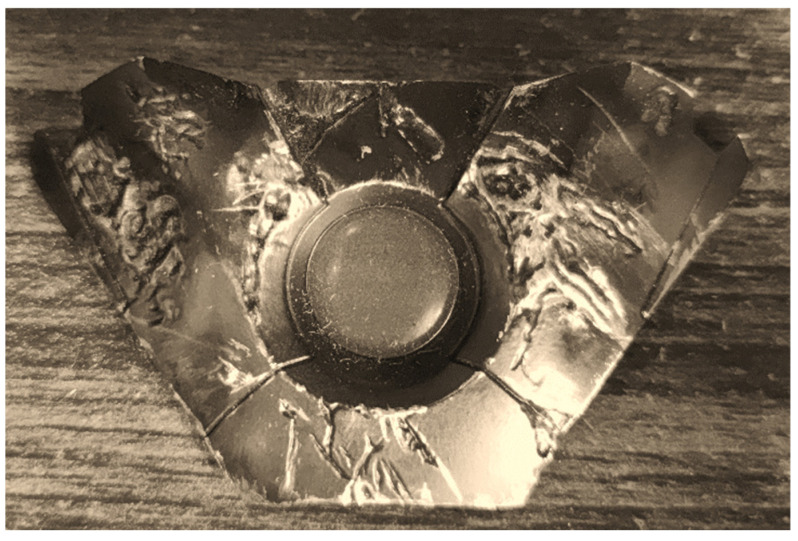
Actual Battle Damage Distribution of Armor.

Figure 5 is the actual situation of the battle damage on the foretold damaged armor; the naked eye can observe several obvious bullet marks, and the purpose of this experiment is to be able to use a more convenient method to detect the position of the damage and the degree of damage to the armor. Then, following much damage, to repair the area of insufficient strength, you need to use subsequent damage assessment and additive manufacturing methods for detection patching.

## 6. Discussion

IMD-based nodal technology was used to attach a thin film to the surfaces of parts. In-mold decoration is an efficient, durable, and cost-effective technique that, unlike traditional surface printing, sets labels or decorations between film and resin. Place the printed film on one side of the mold and inject the molten plastic into the back of the film. The film and plastic are combined into a single unit, or the decoration is embedded in it. Compared with other methods, it can improve the durability of the finished product.

**Figure 6:**
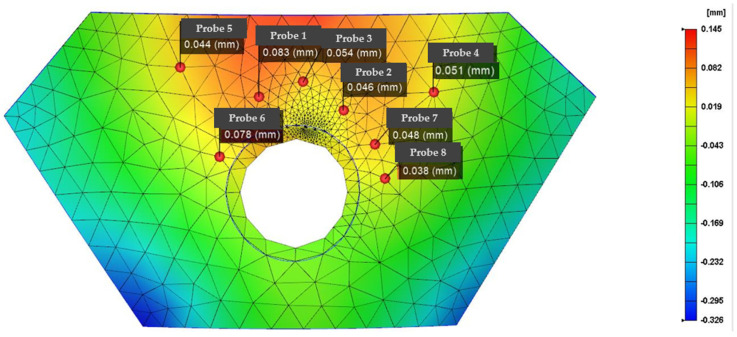
Optimizes the pre-armor die index analysis data graph.

Many parameters are involved in the manufacturing of IMD. If these manufacturing parameters are not set correctly, the finished product can easily become defective. Specifically, IMD defects and parts that need to be improved are concentrated in locations prone to large and small defects from war damage. The error range of node measurement is basically stable between 0.01 and 0.1. According to different node deviations to divide the damage degree, node offset below 0.01 is so small as to be negligible, and offset greater than 0.1 mm represents beyond the repairable range. The main method of improvement is to set various parameters of IMD, adjust the injection-molding parameters, adjust the thin-film material and molding parameters, and analyze the die index results, which are important indicators of the product quality. It is known from experience that the defect location is generally distributed in the position with the smallest thickness of the entire part, and it is also the part where the mold index changes the most. It is necessary to perform factor analysis and improve the defects in the part, as well as examining the IMD analysis results of the armor parts before and after optimization, the mold index before and after the IMD mesh analysis, and the optimization of the film parameters to predict the damage defects in the armor. Figure 6 shows the die indices of the eight nodes that are representative of the damaged area. The red area represents the larger position of the die index, which is more likely to cause damage, the blue area represents a smaller replay index, and the strength is greater than the pit strike ability in the red area.

**Figure 7:**
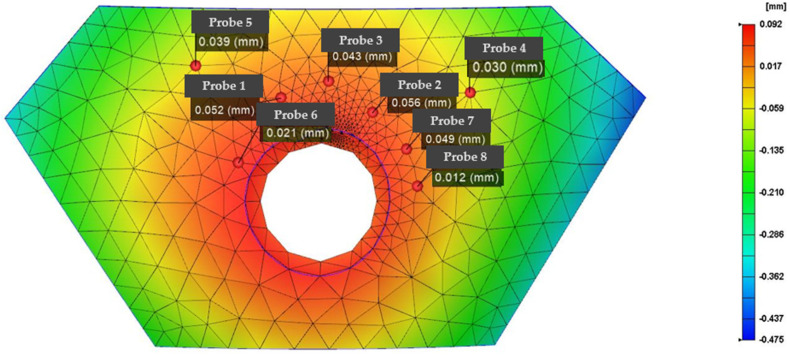
Optimized armor stamping index analysis data graph.

This experiment uses mold flow analysis software to predict the degree of damage and the damage position in the armor and the strength optimization of the vulnerable area by changing the molding parameters; the resulting optimized damage position is shown in the die index in Figure 7. Because in the analysis and prediction, the die node technology proposed based on this experiment is proposed for the first time, and the location with the high stamp index is the area with large damage and vice versa, subsequent verification is required.

Table 1 presents the results of comparing the molding parameter optimization before and after the die index optimization. Except that node 7 failed to have a good optimization effect, the nodes achieved better optimization; the average optimization rate after removing the larger error point reaches 38.7%, and the optimized die index gives the molded armor higher strength, ensuring that the weak position that is prone to damage is reinforced.

**Figure 8:**
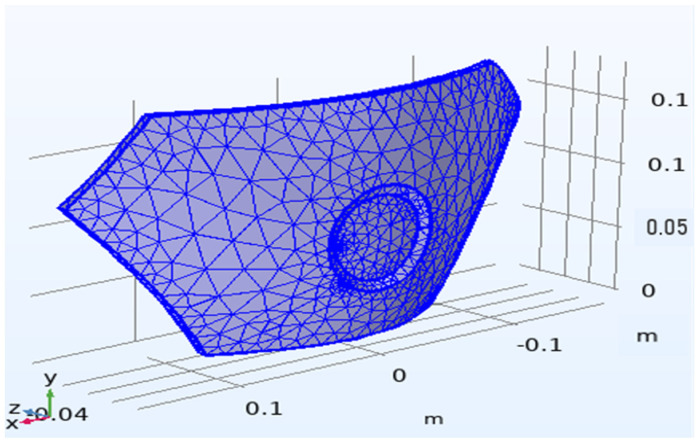
Meshing of Comsol armor in the Johnson-Cook model.

**Figure 9:**
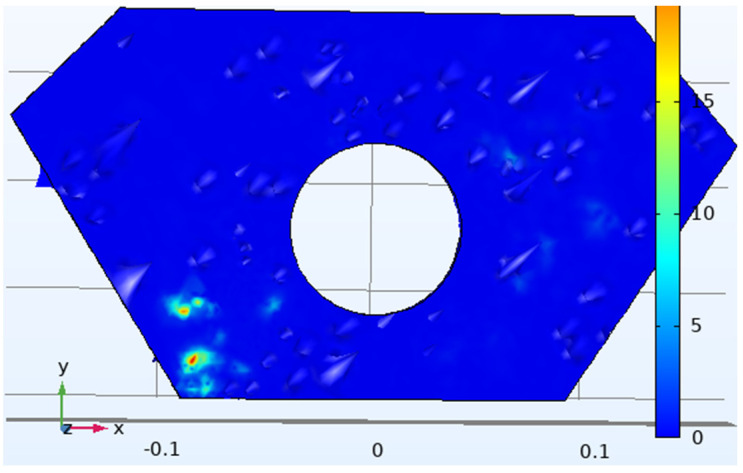
Predicting the outlook of the Comsol armor in the Johnson-Cook model.

In order to verify the location of the damage, we also performed the relevant load-loading model verification of the Johnson–Cook model in Comsol 5.6 software; the meshing diagram of the model is shown in Figure 8. Using this model theory to make assumptions about stress distribution and simulating the load impact when the armor is damaged, a graph of the damage situation of the relevant maximum damaged position is obtained, as shown in Figure 9; then, the results of the mold flow software are compared to verify the accuracy of the node technology.

**Figure 10:**
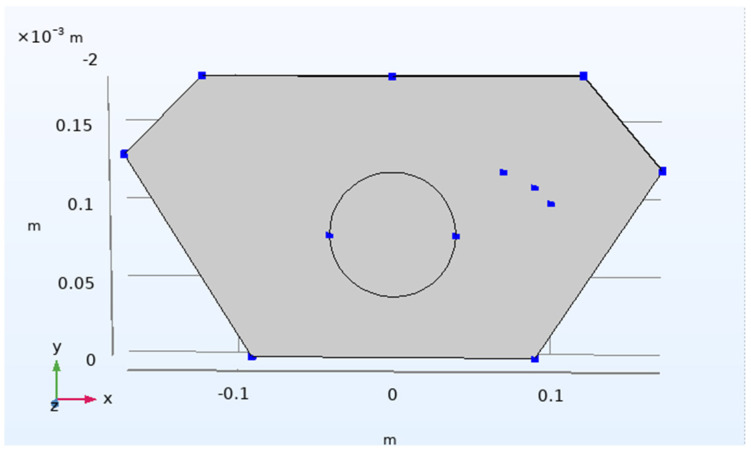
The Comsol Johnson-Cook model predicts a graph of 8 node locations.

**Figure 11:**
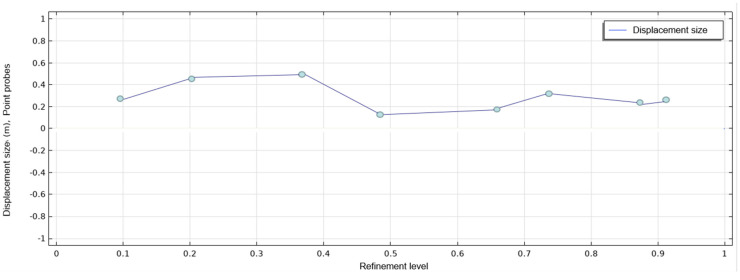
The Comsol Johnson-Cook model predicts a numerical graph of 8 nodes.

In the simulation results from Comsol, the same node position is sampled, eight regional node probes are set up, and the specific sampling position of the probe is shown in Figure 10 to obtain the deformation displacement value required for the experimental results and obtain the data basis required for subsequent analysis. The display results for the probe are shown in Figure 11.

According to the comparison between the Comsol stress simulation results in Table 2 and the matching degree analysis of the die index, except for node 6 and node 8, which failed to meet the convergence requirements well, the nodes reached a match of more than 85%, which proved that the optimization results of the die index for the detection and optimization of battle loss were true and effective. Combined with IMD, the tensile properties, thickness, bending properties, and elongation properties of the armor materials itself were improved. When setting its parameters, pay attention to the influence of the diaphragm shape, the fillet radius of the punch, the blank clamping force, the gap between the punch, and the punch on the drawing depth. In order to avoid the deformation of the product, when designing the film thickness, the wall thickness must be consistent according to the analysis. When solving for stagnation temperatures caused by plastic films, the temperature difference for heat transfer can be calculated with a cooling analysis, and the amount of deformation can be minimized by compensating for the mold temperature on the mold side. Therefore, the die index analysis of IMD film is extended to the defect analysis of the surface of the armor because the die defect in the film is very similar to the surface damage defect in the armor and can be analyzed as the same defect factor; the experimental results also show that this technique can be used for armor damage detection.

**Figure 12:**
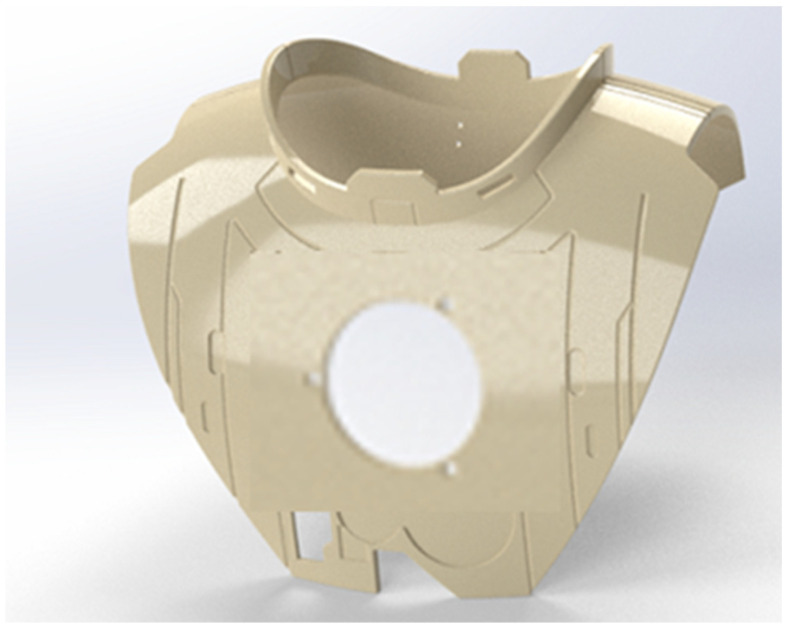
Complete armor diagram after restoration.

The prediction results are in line with the actual requirements to model and predict the material flow behavior of glass fiber using the Johnson–Cook flow stress and the Johnson–Cook failure models. Using these material parameters, nodal damage testing was performed on damaged armor sites with different radii between 0.5 mm and 1 mm under tensile load. This experiment was calculated to predict the degree of level 2 damage. The shape of the repaired armor is shown in Figure 12, which is the result of the filling repair of the damage location using optimization and subsequent polymer glass-fiber PEEK material 3D printing technology.

## 7. Conclusions

In this experiment, we extended the die index analysis of IMD films to the defect analysis of the armor surface because the die defects in the film are very similar to the surface damage defects of the armor and can be analyzed as the same defect factor; therefore, we used this technique for armor damage prediction. The prediction results indicate that the method is in line with the actual requirements for modeling and predicting the material flow behavior of glass fiber using the Johnson–Cook flow stress and the Johnson–Cook failure models. Using these material parameters, nodal damage test ratings were performed on damaged armor sites with different radii between 0.5 mm and 1 mm under tensile load. This experiment calculated and predicted the degree of level 2 damage.

As shown from the degree of optimization of the stamping die index after the repair, the results obtained by the optimization of the method in this experiment and the subsequent filling and repair of the damage location by the 3D printing of the polymer glass-fiber PEEK material are in line with the experimental expectations. Combined with the tensile properties, thickness, bending properties, and elongation properties of the circuit of the IMD film itself, combined with the Johnson–Cook stress model analysis, when setting its parameters, pay attention to the influence of the diaphragm shape, the fillet radius of the punch, the blank clamping force, the gap between the punch and the punch, etc. on the drawing depth. In order to avoid the deformation of the product, when designing the film thickness, the wall thickness must be consistent according to the analysis. When solving for stagnation temperatures caused by plastic films, the temperature difference for heat transfer can be calculated with a cooling analysis, and the amount of deformation can be minimized by compensating for the mold temperature on the mold side.

The main conclusions and contributions of the research and experiments carried out in this paper can be listed as the following three points:

1. The battle loss model of the armor is obtained by scanning the 3D measurement of the actual battle damage armor, and the simulation map of the battle loss area in the mold flow analysis is obtained by node analysis.
2. The specific node value of the die index is analyzed, that is, the battle loss of the vulnerable parts of the armor, so as to further optimize the molding parameters to reinforce the vulnerable position of the armor, and the degree of optimization reaches 38.7%.
3. The structural mechanics simulation module of Comsol is used to further verify the damaged parts of the armor, and the Johnson–Cook stress analysis model is introduced to simulate the boundary load of the damaged position of the armor. The matching degree between the Comsol results and the die index is more than 85%, which verifies the accuracy of the die index analysis method.

## Figures and Tables

**Table 1:** Before-and-After Die Index Optimization Comparison Results.

Number of Nodes	Die Index (mm)	Optimized Die Index (mm)	Optimized Efficiency
1	0.083	0.052	37.3%
2	0.046	0.056	21.7%
3	0.054	0.043	20.3%
4	0.051	0.030	41.1%
5	0.044	0.039	11.3%
6	0.078	0.021	73.1%
7	0.049	0.048	2%
8	0.038	0.012	68.4%
average			38.7%

**Table 2:** Comsol stress simulation results match the die index table.

Number of Nodes	Optimized Die Index (mm)	Comsol Simulation Value (mm)	The Degree of Match
1	0.052	0.046	88.5%
2	0.056	0.059	95.0%
3	0.043	0.048	89.6%
4	0.030	0.031	96.8%
5	0.039	0.042	92.9%
6	0.021	0.044	47.7%
7	0.048	0.047	97.9%
8	0.012	0.030	40%

## Data Availability

Not applicable.

## References

[1] Chen S.-C., Huang S.-T., Lina M.-C., Chien R.-D. (2008). Study on the thermoforming of PC films used for in-mold decoration. Int. Commun. Heat Mass Transf..

[2] Phillips C.O., TClaypole C., Gethin D.T. (2008). Mechanical properties of polymer films used in in-mould decoration. J. Mater. Process. Technol..

[3] Kim G., Lee K., Kang S. (2009). Prediction of the film thickness distribution and pattern change during film insert thermoforming. Polym. Eng. Sci..

[4] Chen S.-C., Li H.-M., Huang S.-T., Wang Y.-C. (2010). Effect of decoration film on mold surface temperature during in-mold decoration injection molding process. Int. Commun. Heat Mass Transf..

[5] Zhang Y., Deng Y.M., Sun B.S. (2009). Injection Molding Warpage Optimization Based on a Mode-Pursuing Sampling Method. Polym. Plast. Technol. Eng..

[6] Martinez A., Castany J., Aisa J. (2011). Characterization of In-Mold Decoration Process and Influence of the Fabric Characteristics in This Process. Mater. Manuf. Process..

[7] Aguiar R., Petel O.E., Miller R.E. (2022). Effect of a Halloysite-polyurethane nanocomposite interlayer on the ballistic performance of laminate transparent armour. Polym. Mater. Part C Open Access.

[8] Abtew M.A., Boussu F., Bruniaux P., Loghin C., Cristian I., Chen Y., Wang L. (2018). Forming characteristics and surface damages of stitched multi-layered para-aramid fabrics with various stitching parameters for soft body armour design. Polym. Mater. Part A Appl. Sci. Manuf..

[9] Mawkhlieng U., Majumdar A., Laha A. (2020). A review of fibrous materials for soft body armour applications. RSC Adv..

[10] Crouch I.G. (2021). Critical interfaces in body armour systems. Def. Technol..

[11] Islam M.K., Hazell P.J., Escobedo J.P., Wang H. (2021). Biomimetic armour design strategies for additive manufacturing: A review. Mater. Des..

[12] Khare S., Kumar K., Choudhary S., Singh P.K., Verma R.K., Mahajan P. (2020). Determination of Johnson–Cook Material Parameters for Armour Plate Using DIC and FEM. Met. Mater. Int..

[13] Li X., Vaz M.A. (2021). Analytical estimation on the number of bending cycles to initiate armour wires lateral buckling in flexible pipes. Ocean. Eng..

[14] Bhat A., Naveen J., Jawaid M., Norrrahim M.N.F., Rashedi A., Khan A. (2021). Advancement in fiber reinforced polymer, metal alloys and multi-layered armour systems for ballistic applications—A review. J. Mater. Res. Technol..

[15] Chang H., Zhang G., Sun Y., Lu S. (2022). Non-Dominant Genetic Algorithm for Multi-Objective Optimization Design of Unmanned Aerial Vehicle Shell Process. Polymers.

[16] Chang H., Zhang G., Sun Y., Lu S. (2022). Using Sequence-Approximation Optimization and Radial-Basis-Function Network for Brake-Pedal Multi-Target Warping and Cooling. Polymers.

[17] Lee S.Y., Jang S.H., Lee H.K., Kim J.S., Lee S.K., Song H.J., Jung J.W., Yoo E.S., Choi J. (2020). The development and investigation of highly stretchable conductive inks for 3-dimensional printed in-mold electronics. Org. Electron..

[18] Gong Y., Cha K.J., Park J.M. (2020). Deformation characteristics and resistance distribution in thermoforming of printed electrical circuits for in-mold electronics application. Int. J. Adv. Manuf. Technol..

[19] Guo W., Yu Z., Wei W., Meng Z., Mao H., Hua L. (2021). Effect of film types on thermal response, cellular structure, forming defects and mechanical properties of combined in-mold decoration and microcellular injection molding parts. J. Mater. Sci. Technol..

[20] Wu C.H., Li J.H. (2020). The use of 3D in-mold decoration technology to form a film with printed circuits. Polym. Eng. Sci..

[21] Liu X., Li D., Fukutani H., Trudeau P., Khoun L., Mozenson O., Sampson K.L., Gallerneault M., Paquet C., Lacelle T. (2021). UV-Sinterable Silver Oxalate-Based Molecular Inks and Their Application for In-Mold Electronics. Adv. Electron. Mater..

[22] Moayyedian M., Dinc A., Mamedov A. (2021). Optimization of Injection-Molding Process for Thin-Walled Polypropylene Part Using Artificial Neural Network and Taguchi Techniques. Polymers.

[23] Chang H.-J., Zhang G.-Y., Su Z.-M., Mao Z.-F. (2021). Process Prediction for Compound Screws by Using Virtual Measurement and Recognizable Performance Evaluation. Appl. Sci..

